# Analysis of the IGS rRNA Region and Applicability for *Leishmania* (*V*.) *braziliensis* Characterization

**DOI:** 10.1155/2020/8885070

**Published:** 2020-10-06

**Authors:** Tayná C. de Goes, Rayana C. S. de Morais, Maria G. N. de Melo, Rômulo P. e Silva, Antônio M. Rezende, Milena de Paiva-Cavalcanti

**Affiliations:** Aggeu Magalhães Institute, Av. Moraes Rego, Cidade Universitária, 50670-420 Recife, PE, Brazil

## Abstract

The causative species is an important factor influencing the evolution of American cutaneous leishmaniasis (ACL). Due to its wide distribution in endemic areas, *Leishmania* (*V*.) *braziliensis* is considered one of the most important species in circulation in Brazil. Molecular targets derived from ribosomal RNA (rRNA) were used in studies to identify *Leishmania* spp.; however, the Intergenic Spacer (IGS) region has not yet been explored in parasite species differentiation. Besides, there is a shortage of sequences deposited in public repositories for this region. Thus, it was proposed to analyze and provide sequences of the IGS rRNA region from different *Leishmania* spp. and to evaluate their potential as biomarkers to characterize *L. braziliensis*. A set of primers was designed for complete amplification of the IGS rRNA region of *Leishmania* spp. PCR products were submitted to Sanger sequencing. The sequences obtained were aligned and analyzed for size and similarity, as well as deposited in GenBank. Characteristics of the repetitive elements (IGSRE) present in the IGS rRNA were also verified. In addition, a set of primers for *L. braziliensis* identification for qPCR was developed and optimized. Sensitivity (*S*), specificity (*σ*), and efficiency (*ε*) tests were applied. It was found that the mean size for the IGS rRNA region is 3 kb, and the similarity analysis of the sequences obtained demonstrated high conservation among the species. It was observed that the size for the IGSRE repetitive region varies between 61 and 71 bp, and there is a high identity between some species. Fifteen sequences generated for the IGS rRNA partial region of nine different species were deposited in GenBank so far. The specific primer system for *L. braziliensis* showed *S* = 10 fg, *ε* = 98.08%, and log*σ* = 10^3^ for *Leishmania naiffi*; log*σ* = 10^4^ for *Leishmania guyanensis*; and log*σ* = 10^5^ for *Leishmania shawi*. This protocol system can be used for diagnosis, identification, and quantification of a patient's parasite load, aiding in the direction of a more appropriate therapeutic management to the cases of infection by this etiological agent. Besides that, the unpublished sequences deposited in databases can be used for multiple analyses in different contexts.

## 1. Introduction

American cutaneous leishmaniasis (ACL) is a zoonosis caused by multiple species of protozoa, which belong to the genus *Leishmania* and family Trypanosomatidae [1]. So far, 11 dermotropic species of those parasites were identified in the new world: eight of them belong to the *Viannia* subgenus and three belong to the *Leishmania* subgenus. The evolution of this disease may range from spontaneous remission of lesions to progression of clinical manifestations, which may be diverse, and it depends on several factors, including the host immune condition and protozoan species involved [2].

In Brazil, due to its wide distribution over all areas where ACL is endemic, *Leishmania* (*Viannia*) *braziliensis* is considered the species that has the most clinical-epidemiological significance. The infection caused by this species tends to progress to a simpler form of the disease, the localized cutaneous leishmaniasis (LCL); however, when the immune system is particularly stimulated, the mucocutaneous (ML) form may occur. The ML disease induces a CD4 cellular response profile, predominantly of the Th1 type, which increases hypersensitivity. This feature makes ML a difficult disease to treat, sometimes leading to extreme tissue damage [3].

Early diagnosis combined with species identification is an important feature to support disease control programs. It can help in indicating the most efficient treatment, which may also vary according to a patient's clinical manifestations [4]. In view of the difficulties in carrying out Multilocus Enzyme Electrophoresis (MLEE), the gold standard technique for species identification, many studies have demonstrated the efficacy of the quantitative Real-Time PCR (qPCR) in identifying the species of parasites due to its high accuracy and sensitivity [5–7].

The most used targets to perform PCR assays that are aimed at diagnosis and/or species identification are those which are derived from genes that encode ribosomal RNAs (rRNA). These genes have interspersed sequences, which are divergent, in general, among and within species, such as the region known as the Internal Transcribed Spacer (ITS) [8, 9], and they also have conserved coding sequences such as the Small Subunit (SSU rRNA) [10].

Other targets already described for species characterization are the kinetoplast DNA (kDNA) minicircles [11], glycoprotein 63 (GP63) [12], cytochrome b (Cyt b) [13], the heat shock protein (HSP) [14], and the glucose-6-phosphate-dehydrogenase (G6PD) [15] genes.

Nevertheless, in accordance with some studies involving the targets described above, there is difficulty in differentiating the species belonging to the subgenus *Viannia* due to the genetic similarity among them [16, 17].

The rRNA Intergenic Spacer (IGS rRNA) region was not investigated so far regarding identification of the *Leishmania* species, although it has already been used in other organisms [18–23]. This region has a size that varies between 4 and 12 kb due to one repeated element named as the IGSRE region (IGS rRNA repeated region), which has between 16 and 275 copies; each one of them is around 62 bp, and this is where the highest amount of the regulatory elements can be found, which are crucial for rRNA gene transcription [24–26].

The existing studies regarding to the IGS rRNA were conducted especially for *Leishmania* subgenus, demonstrating a high variability related to the size, sequence, and number of repetitions [25, 26]. However, there are no studies related to the characterization of such region regarding the species of the subgenus *Viannia*. Moreover, just a few sequences are available in genomic databases.

In view of this, the present work is aimed at analyzing and providing sequences of the IGS rRNA region from different *Leishmania* spp., and also evaluating their potential as biomarkers to characterize *L. braziliensis*, through qPCR.

## 2. Materials and Methods

### 2.1. Primer Design for *Leishmania* Spp. IGS rRNA Region Amplification

**Figure 1:**
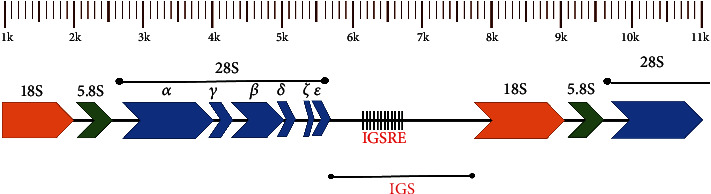
Forming subunits of the *Leishmania* spp. ribosomal RNA. Repeating unit of an RNA locus, showing the 18S (orange), 5.8S (green), and 28S (blue) regions. In the intergenic space (IGS) between the repetition units, there is an element of repetition called IGSRE. It is estimated that the IGS region size is between 2 and 3 kb.

Based on *Leishmania major* sequences that encode the 18S (LmjF.27.rRNA.03) and the 28S rRNA (LmjF.27.rRNA.46), which are available in the TriTrypdb [27], a pair of primers was designed: a reverse primer at the beginning of 18S, and a forward primer at the end of 28S (LSU*ε*). This pair of primers was used for the complete amplification of the IGS rRNA region (see Figure 1) of different *Leishmania* species. The primers were designed using the Primer Express Software (Applied Biosystems, version 2.0). Through the distance between the reference sequences (LmjF.27.rRNA.03 and LmjF.27.rRNA.46), the product size of the amplicon was estimated. The designed primers were also submitted to the BLAST*n* platform [28] to search the sequences isolated from the IGS rRNA region within sequences of the Chromosome 27 of *Leishmania* spp., and to determine the sizes and sequences for each species, which were used as references for *in silico* alignment.

### 2.2. PCR of IGS rRNA Region for Different *Leishmania* Species

Using the designed set of primers and genomic DNA samples from reference strains of 11 *Leishmania* species (see Table 1) obtained from the Leishmaniose Reference Services of the Aggeu Magalhães Institute, a conventional PCR was performed using the following cycling and reaction conditions: Taq DNA polymerase recombinant enzyme (Invitrogen™, CA, USA), with both primers at a concentration of 10 *μ*M; 10x PCR buffer; 2 mM of dNTPs; 25 mM of MgCl_2_; 2 *μ*L of DNA template at a concentration of 10 ng; and type I water. Thus, the complete final volume is 25 *μ*L. Cycling conditions were 94°C/2 minutes (initial denaturation); 10 cycles of 94°C/30 seconds (denaturation), 64°C/30 seconds (primer hybridization), and 68°C/2 minutes and 30 seconds (DNA extension); and followed by 35 cycles of 94°C/30 seconds (denaturation), 54°C/30 seconds (primer hybridization), and 72°C/2 minutes and 30 seconds (DNA extension).

The reagent mix without the DNA sample was used as a negative control. Temperature cycling was performed using the Eppendorf Mastercycler gradient equipment.

The PCR products were analyzed through agarose gel electrophoresis. The amplicons with the closest sizes to those estimated through *in silico* analysis were excised from the gel and submitted to purification and sequencing.

### 2.3. Sequencing of PCR Products

The specific amplified fragments extracted from the agarose gel obtained after the PCR reaction were purified through the PureLink Quick Gel Extraction and PCR Purification Combo kit (Invitrogen, Life Technologies™, CA, USA) and submitted to Sanger sequencing using the designed primers (Section 2.1) and the BigDye Terminator Cycle Sequencing Kit (version 3.1) (Applied Biosystems, CA, USA), in an automatic sequencer (ABI 3500xL Genetic Analyzer). The sequences were determined in two directions for each species. Each fragment was sequenced twice on average.

### 2.4. *In Silico* Sequence Analysis and Alignment

Using the Chromas software (version 2.6.6), the Phred score [29] was verified for sequences obtained by the Sanger sequencing assay. Afterwards, these sequences were analyzed by BLAST*n* to verify the coverage and identity with *Leishmania* spp. Chromosome 27 sequences available on GenBank. Using the MAFFT (Multiple Alignment using Fast Fourier Transform) algorithm, through the softwares AliView (version 1.18.1) and BioEdit (version 7.2.6), alignments between the studied and the reference sequences were performed (Section 2.1). Through the alignments, similarities and conservation level between the IGS rRNA region and species were verified.

Moreover, it was possible to localize, by visual identification, the IGSRE region for some species. Afterwards, a comparison between species and subgenus, related to size and quantity of repetitions, was performed in this region.

The sequences obtained in this work were deposited in the GenBank database through the BankIt/NCBI platform [30].

### 2.5. Development and Optimization of Primer System for *L. braziliensis* Identification

From the performed alignment, a primer system that was able to identify only the *L. braziliensis* species was designed. The system design was performed using the software Primer Express (Applied Biosystems, version 2.0).

To define the detection limit, serial dilutions (factor 10) of genomic DNA from *Leishmania* spp. reference strains in quantities of 1 fg to 1 ng were prepared, and as negative control, a sample without DNA was used. Detection limits were defined in order to be applied in SYBR® Green-based qPCR, using QuantStudio™ (Applied Biosystems®, CA, USA) equipment. All samples were performed in duplicate, and the following conditions were employed for the experiments: 95°C/15 s (denaturation) and 60°C/1 min (primer hybridization), at 40 cycles.

The sensitivity (*S*) of the assay was defined as the lowest DNA concentration which promoted amplification of the target. The efficiency (*ε*) was determined by the following formula: *ε* = (10^−1/slope^) − 1. In order to evaluate the specificity (*σ*) for *L. braziliensis*, five other *Leishmania* spp. (*L. naiffi*, *L. guyanensis*, *L. shawi*, *L. infantum*, and *L. amazonensis*) and *Trypanosoma cruzi* (strain Y) genomic DNA were used as test samples. The specificity was defined by *σ* = (1 + *ε*)^Δ*C*_t_^, where *ε* stands for amplification efficiency and Δ*C*_t_ is the difference in threshold cycle (*C*_t_) values of the target and test samples [30].

Analysis and recording of results of the experiments were carried out using the QuantStudio™ Design and Analysis Software (version 1.4.3).

## 3. Results

### 3.1. Primer Design for *Leishmania* Spp. IGS rRNA Region Amplification

The designed primers based on *L. major* sequences (Section 2.1) displayed a profile demonstrated in Table 2.

From a GenBank search, eight species were found to have complete or partial sequences available for Chromosome 27: *Leishmania infantum* (CP027826. 1), *Leishmania mexicana* (FR799580.1), *L. major* (FR796423.1), *Leishmania amazonensis* (AF060493.2), *Leishmania donovani* (CP029526.1), *Leishmania peruviana* (LN609266.1), *Leishmania panamensis* (CP009396.1), and *L. braziliensis* (FR799002.1). The designed primers (Section 2.1) were localized in these sequences, and only their products were isolated, that is, the IGS rRNA regions, which were used as reference.

The searching for oligonucleotides in BLAST*n* demonstrated the possibility for isolating the IGS rRNA region from five species wherein the two primers were found. A great variety related to the quantity and size of the region among species was observed. The average size obtained for these sequences was approximately 3 kb (see Table 3).

**Figure 2:**
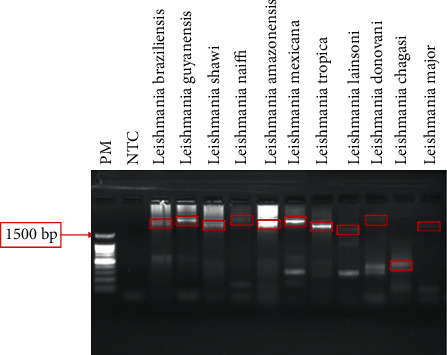
Agarose gel (1.0%) showing PCR reaction result for IGS rRNA region amplification of different *Leishmania* spp. IGS—Intergenic Spacer; rRNA—ribosomal RNA; bp—base pairs; PM—marker 100 bp (Promega); NTC—nontemplate control. Boxes show bands extracted for purification.

The species used for the molecular test showed a diverse band pattern (see Figure 2), in which the majority demonstrated at least one band size compatible with the estimated *in silico* (3 kb).

### 3.2. Capillary Sequencing of the IGS rRNA Region

A length average of 837.3 bases for sequenced species using the 28SF primer and a length average of 846.4 bases for sequenced species using the 18SR primer were obtained. Nine out of eleven species demonstrated a good sequence quality (Phred score: ≥30) for the reverse primer, and six for the forward primer (see Table 4). Alignment of all the sequences obtained from the 28SF and 18SR primers separately in each species was performed. It was observed that a considerable similarity among the species was presented. The sequences obtained from the 18SR primer achieved a better quality, in which the Phred scores were above 30, thus enabling the analysis of all of them by BLAST*n* (see Table 5).

It was observed that in the alignment of the sequences obtained from the 18SR primer, it was indicated that the sequence of *L. braziliensis* was shown to be distinct from those belonging to the different species studied, thus demonstrating that IGS rRNA is a promising region for the unique identification of this species.

### 3.3. *In Silico* Analysis of the IGSRE Region

After capillary sequencing, the repeated IGSRE region was found in sequences from the *L. shawi*, *L. naiffi*, and *L. amazonensis* species. Furthermore, after analysing the complete Chromosome 27 sequences, it was possible to find such region in *L. braziliensis*, *L. peruviana*, *L. donovani*, *L. major*, and *L. mexicana*.

It was observed that the IGSRE from *L. shawi*, *L. naiffi*, and *L. peruviana* species are similar. IGSRE is also similar between *L. amazonensis* and *L. mexicana* (see Table 6).

Likewise, variation in the amount of repetitions in different IGS rRNA regions of the same species was observed (see Table 7).

### 3.4. Depositing Sequences to GenBank

The sequences acquired in this work were placed in the GenBank database under access numbers MK493617 and MK493618 for *L. amazonensis*, MK493607 and MK493608 for *L. braziliensis*, MK493615 and MK493616 for *L. lainsoni*, MK493613 and MK493614 for *L. guyanensis*, MK493609 and MK493610 for *L. shawi*, MK493620 for *L. tropica*, MK493619 for *L. mexicana*, MK493621 for *L. donovani*, and MK493611 and MK493612 for *L. naiffi*.

### 3.5. Design of Specific Primers for *L. braziliensis*

Based on *L. braziliensis* IGS rRNA sequence, a specific set of primers was designed for this species, in order to be applied in SYBR® Green-based qPCR. Their general characteristics are presented in Table 8.

The amplification product of 148 bp from primers Lb/IGS F and R was sequenced and submitted to BLAST*n* analysis. Full coverage and 100%, 99%, and 90% identity with *L. braziliensis*, *L. peruviana*, and *L. panamensis* were demonstrated, respectively.

### 3.6. Analytical Sensitivity and Specificity for the Lb/IGS System

**Figure 3:**
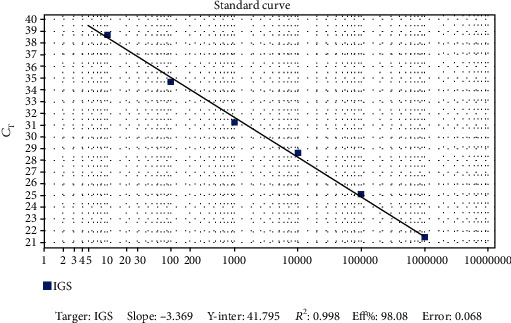
Reaction efficiency with the Lb/IGS system shown in the standard curve. Standard curve resulting from the amplification reaction of *Leishmania braziliensis* (Lb) DNA in six points or concentrations, demonstrating a detection limit of up to 10 fg for this specie. On the ordinate axis is the threshold cycle or cut-off point (*C*_t_), and on the abscissa axis is the amount of template DNA in fentograms (fg). IGS—Intergenic Spacer; rRNA—ribosomal RNA.

The designed system was able to amplify *L. braziliensis* DNA up to 10 fg for the reaction, which is equivalent to 0.13 parasites, and the reaction efficiency was 98.08% (see Figure 3). There was a cross reaction with other species belonging only to the *Viannia* subgenus, with the specificity calculation being applied then, which demonstrated that the system is around 10^3^-fold (*σ* = 827.4), 10^4^-fold (*σ* = 9, 532.7), and 10^5^-fold (*σ* = 67, 858.4) more specific for *L. braziliensis* than for *L. naiffi*, *L. guyanensis*, and *L. shawi*, respectively.

## 4. Discussion

There is a consensus related to the need for distinction among the *Leishmania* species which are harmful to man. This is due to the strong influence presented by different species in clinical manifestation developed in individuals. Thus, the development of efficient diagnostic methods and the discovery of suitable biomarkers able to detect and identify *Leishmania* species are necessary in order to carry out therapeutic approaches that are specie-specific, this being a vital aspect to improve the control programs related to infection caused by *Leishmania* sp. [31, 32].

A greater understanding acquired currently about *Leishmania* sp. genome brought considerable breakthroughs, which were converted into modern diagnostic methods. Several molecular targets have already been studied in order to find one that would be able to categorize the *Leishmania* species [6, 7]. The variation of the results among the existing methodologies lies in the inconsistency in patterns according to the markers used, the possibility of mixed infection, and the similarity between the species, especially those belonging to the subgenus *Viannia*. [15, 16].

Ribosomal RNA (rRNA) derivatives are among the most analyzed molecular targets for the detection of *Leishmania* sp., where the *Small Subunit* (SSU rRNA) stands out, due to their high degree of conservation [10]. For species distinction, the Internal Transcribed Spacer (ITS) is evident. It was already shown to be able to identify *Leishmania* spp. at the complex level, but it was able to identify only *Leishmania aethiopica*, *Leishmania tropica*, *Leishmania major*, and *Leishmania turanica* at the species level [8, 15, 33–35].

The Intergenic Spacer of rRNA (IGS rRNA) demonstrated suitable sequences for differentiation between bacterial species *Bacillus grandii* and *Bacillus atticus*, which are closely related [22], besides trypanosomatids such as *Trypanosoma cruzi* and *Trypanosoma congolense* [18–20]. However, there are no studies reporting the use of such region for the diagnosis or characterization of the *Leishmania* species [7]. In fact, there is a lack of studies focusing on this region in general, it being characterized only for the *L. major* [26], *L. amazonensis* [36], *L. tarentolae*, and *L. hoogstraali* species [25, 37]. There are no detailed studies for the *Viannia* subgenus.

In the present study, a set of primers flanking the IGS rRNA region was designed to fully amplify it. The IGS rRNA region size was different among the five species, which made it possible to delimit this region using the 28SF and 18SR primers, ranging approximately from 2 to 4 kbp (Table 3), diverging from literature data, which show that the minimum size for this region is about 4 kbp [25]. The repetitive IGSRE region found in the present study has the same size and sequence as those previously published for *L. major*, *L. donovani*, and *L. amazonensis* species [26, 36]. Furthermore, we highlight the presence of IGSRE in the sequences of five other species, with a total variation of 6-265 in the number of copies; so far this variation was between 16 and 275 repetitions [26].

The complete sequencing of several *Leishmania* sp. genomes opened a new era related to the diagnosis and identification of causative species of leishmaniasis. Nine species have their complete genome published, but from these, only two are from the *Viannia* subgenus [38–45]. In the present study, part of the IGS rRNA region from eleven different species of *Leishmania* was sequenced, including five species of the *Viannia* subgenus. Among the six species belonging to the *Leishmania* subgenus, the one included in this analysis (*L. infantum*/*chagasi*) does not have its sequence published for such region.

In some cases, the use of methods that generate higher readings, such as Sanger sequencing, may be more suitable for repetitive region analysis [45]. In the meantime, with the results obtained in this work after Sanger sequencing, it was not possible to assemble a consensus sequence, due to the long region size and reading limitation of capillary sequencing.

Currently, there is no unique method to diagnose all of the pathogenic *Leishmania* species [7]. Sequencing-derived techniques (e.g., Multilocus Sequence Typing (MLST)) and PCR (e.g., the Real-Time quantitative PCR (qPCR)) come as alternatives to Multilocus Enzyme Electrophoresis, the gold standard to characterize *Leishmania* spp., which cannot be widespread due to some limitations [6].

A high conservation of the IGS rRNA region was observed, which demonstrates that it could not be applied as a target for characterization at species level. Nevertheless, after the alignment analysis, it was possible to observe a region in which the nucleotide sequence could be used to distinguish *L. braziliensis* species from others of the subgenus *Viannia*; *L. braziliensis* is the etiological agent of ACL with greater distribution in Brazil and in the Americas [2], and a molecular tool able to accurately detect and differentiate this species would be very welcome. The infections caused by *L. braziliensis* correspond to lesions with scarce parasites. In this case, qPCR might be useful to confirm the diagnosis, besides evaluating the parasitic load, providing advantages once infections caused by this species may evolve to mucocutaneous form, which requires differentiated clinical and therapeutic management [46].

Despite the high coverage and identity shown in the *in silico* analysis of the primers of the Lb/IGS system, as only Lb had 100% cob/id, we decided to carry out the experiments, with a proposal for the possible use of hybridization probes to increase specificity. The Lb/IGS primer system presented a satisfactory performance in optimization tests and exhibited a high sensitivity for the detection of *L. braziliensis* in assays with 98.08% analytical efficiency. These results showed a higher efficiency when compared to other similar studies [10, 46]. The cross reaction obtained only for species of the *Viannia* subgenus emphasizes the high genetic similarities among them. However, since the concentrations detected for the species that were not the target of the system were higher than that detected for *L. braziliensis*, it was possible to differentiate them by calculating specificity.

Finally, the availability of the sequences in genomic databases, such as GenBank, enables a wide view of the information generated in the present work and enables new studies to be generated on this underexplored region, such as the development or improvement of molecular diagnostic tools that are aimed at species differentiation, as carried out herein. Besides, the availability of such sequences may boost the execution of studies to acquire a better understanding related to the genome of causative species of ACL, as well as their peculiarities.

## 5. Conclusions

Despite the high conservation for this region, the *L. braziliensis* sequence appeared to distinct and became the target of a set of primers for unique identification of this species. Through the protocol based on optimized quantitative Real-Time PCR, the system can be used for diagnosis, identification, and quantification of a patient's parasite load, supporting and guiding a more appropriate therapeutic approach for cases of infections caused by this etiological agent.

## Figures and Tables

**Table 1:** Codes of different reference strains used in conventional and real-time PCR assays.

Species	Strain code
*Leishmania braziliensis*	MHOM/BR/1975/M2903
*Leishmania guyanensis*	MHOM/BR/1975/M4147
*Leishmania shawi*	IWHI/BR/1999/M17904
*Leishmania naiffi*	MDAS/BR/1990/M5533
*Leishmania lainsoni*	MHOM/BR/1981/M6426
*Leishmania amazonenses*	IFLA/BR/1967/PH8
*Leishmania mexicana*	MHOM/BZ/1982/BEL 21
*Leishmania tropica*	MHOM/SU/1958/strain OD
*Leishmania major*	MRHO/SU/1959/P-strain
*Leishmania donovani*	MHOM/ET/1967/HU3
*Leishmania infantum*/*chagasi*	MHOM/TN/1980/IPT1
*Trypanosoma cruzi*	Y strain

**Table 2:** Characteristics of primers designed for *Leishmania* spp. IGS rRNA region amplification.

Genome location	Primer	Size	% C/G	*T*_m_
*L. major* region 28S rRNA end	28S F 5′-GAGGCCTGAAATTTCATGCTC-3′	21 bases	47.62%	62°C
*L. major* region 18S rRNA start	18S R 5′-GATCTGGTTGATTCTGCCAG-3′	20 bases	50%	60°C

IGS—Intergenic Spacer; rRNA—ribosomal RNA; % C/G—cytosine/guanine percentage; *T*_m_—melting temperature.

**Table 3:** Search results for BLAST*n* 28SF and 18SR primers.

Primers found	Species/ID	Quantity of IGS rRNA regions found	Average size of IGS rRNA (kb) regions
28SF and 18SR	*Leishmania donovani*/CP022642	2	3.22
	*Leishmania peruviana*/LN609266	1	1.39
	*Leishmania mexicana*/FR799580	2	2.03
	*Leishmania major*/FR796423	6	4.41
	*Leishmania amazonensis*/AF060493	1	2.5
28SF	*Leishmania braziliensis*/FR799002	—	—

Legend: IGS—Intergenic Spacer; rRNA—ribosomal RNA; ID—NCBI sequence identification code; kb—kilobases; F—forward; R—reverse.

**Table 4:** Number of bases sequenced per *Leishmania* species after capillary sequencing.

Specie	Size (bases)	
	28SF	18SR
*Leishmania braziliensis*	371	934
*Leishmania guyanensis*	921	524
*Leishmania shawi*	1026	942
*Leishmania naiffi*	1008	1012
*Leishmania lainsoni*	756	763
*Leishmania amazonensis*	936	1000
*Leishmania mexicana*	—	981
*Leishmania tropica*	—	928
*Leishmania donovani*	—	541

Legend: F—forward; R—reverse.

**Table 5:** BLAST*n* analysis of *Leishmania* species sequenced from 28S F and 18S R primers.

Species	BLAST sequence per primer			
	28S F (cov%/id%)	Species with greater similarity	18S R (cov%/id%)	Species with greater similarity
*Leishmania braziliensis*	100/99	*Leishmania peruviana*	98/99	*L. panamensis*
*Leishmania guyanensis*	86/99	*Leishmania panamensis*	22/99	*L. panamensis*
*Leishmania shawi*	99/97	*L. peruviana*	95/87	*L. braziliensis*
*Leishmania naiffi*	99/96	*L. peruviana*	99/89	*L. peruviana*
*Leishmania lainsoni*	98/97	*L. panamensis*	45/74	*L. braziliensis*
*Leishmania amazonensis*	100/97	*L. amazonensis*	100/99	*L. amazonensis*
*Leishmania mexicana*	—	—	100/99	*L. mexicana*
*Leishmania tropica*	—	—	100/96	*L. donovani*
*Leishmania donovani*	—	—	63/100	*L. donovani*

Legend: F—forward; R—reverse; BLAST*n*—Basic Local Alignment Search Tool nucleotide; cov%—coverage percentage; id%—identity percentage.

**Table 6:** Size of the IGSRE repetitive regions of different *Leishmania* spp.

Species	IGSRE	Size (bp)
Lb	CTGTGGCGGCCCCCTTTGTTATGTTGTTCAGAAGCACATCCGTCGATTCCTTTTGTCTCCTTTCCGATGCC	71
Lp	GAAGCACCACACTGCACAGCTGCGCATCGCCACCAAGACGGGTCAAAAGCAGCGGAACGCA	61
Ls	GAAGCACCACACTGCACAGCTGCGCATAGCCGCCAAGACGGGTCAAAAGCAGCGGAACGCA	61
Ln	GAAGCACCACACTGCACAGCTGCGCATAGCCGCCAGGACGGATCAAAAGCAGCGGAACGCA	61
La	CCCAGTACTTGGACATTCCCTTTCGCCACGCGCAGGGCCGCTGGAGGCGCACTCCTTCGCGT	62
Lmx	CCCAGTACTTGGACATTCCCTTTCGTCACGCGCAGGGCCGTCGGAGGCGCACTGGTTTGCGT	62
Ld	TTTTCGCTGCGAGGGAGACCCTCGCGGGAGCATTGCTTCGCGCCCCAGTACTGGAACATTCCCG	64
Lmj	CCCCAGTAGTCGAGCATTTGCGTTTCGCCACGAGGGAGGCTCTTGCAGGAGCGTTGTGTCGCG	63

Legend: IGS—Intergenic Spacer; rRNA—ribosomal RNA; Ld—*Leishmania donovani*; Lb—*Leishmania braziliensis*; Lmj—*Leishmania major*; Lmx—*Leishmania mexicana*; Lp—*Leishmania peruviana*; Ls—*Leishmania shawi*; Ln—*Leishmania naiffi*; La—*Leishmania amazonensis*; bp—base pairs.

**Table 7:** Size of the IGSRE repetitive region of different *Leishmania* spp.

Species	Number of repetitive regions (IGSRE) inserted in each IGS rRNA region					
	*1st*	*2nd*	*3rd*	*4th*	*5th*	*6th*
*Leishmania major*	**8**	**12**	**15**	**37**	**28**	**10**
*Leishmania braziliensis*	**6**	**14**	**39**	**60**	—	—
*Leishmania donovani*	**23**	**20**	—	—	—	—
*Leishmania mexicana*	**34**	**30**	—	—	—	—
*Leishmania peruviana*	**265**	—	—	—	—	—

The number of repetitions of the IGS rRNA region inserted in Chromosome 27 is shown in italics. The quantity of repetitions of the IGSRE region for each IGS rRNA region, by *Leishmania* species, is shown in bold numbers.

**Table 8:** Set of primers specific for *Leishmania braziliensis* targeting the IGS rRNA region.

Genome location	Primers	Size	% C/G	*T*_m_
IGS rRNA	Lb/IGS F 5′-CGCACAAAACACACACTGGT-3′	20 bases	50%	59.8°C
IGS rRNA	Lb/IGS R 5′-TTCATGCTCAGGGACACACTC-3′	21 bases	52%	60°C

Legend: IGS—Intergenic Spacer; rRNA—ribosomal RNA; Lb—*Leishmania braziliensis*; F—forward; R—reverse; % C/G—percentage of cytosine/guanine; *T*_m_—melting temperature.

## Data Availability

The dataset supporting the conclusions of this article is included within the article (and its additional file).
